# Peritoneal Inclusion Cysts Presenting as a Femoral Hernia: A Case Report

**DOI:** 10.7759/cureus.108785

**Published:** 2026-05-13

**Authors:** Chun Kit Kenny Ling, Tamara Enthoven, Mohamed Ibrahim

**Affiliations:** 1 General Surgery, Hereford County Hospital, Hereford, GBR; 2 Public Health and Primary Care, University of Cambridge, Cambridge, GBR

**Keywords:** femoral hernia, groin mass, hydrocele of the canal of nuck, peritoneal inclusion cysts, ultrasound imaging

## Abstract

Peritoneal inclusion cysts (PICs) are rare benign mesothelial cystic proliferations, typically occurring in women with prior abdominopelvic pathology. We report a case of a woman in her 70s presenting with an irreducible groin mass, found to have a concurrent fatty indirect inguinal hernia and femoral hernia containing multiloculated PICs in the absence of known risk factors, including prior abdominopelvic surgery or inflammation. Preoperative ultrasound suggested a hydrocoele of the canal of Nuck.

Intraoperative exploration revealed a multiloculated cystic lesion within the femoral canal containing straw-coloured fluid without internal communication. The lesion was excised, and histopathology confirmed PICs without malignancy.

This case highlights an atypical presentation of PICs in an older patient without predisposing factors and demonstrates the potential for diagnostic misinterpretation on imaging. Clinicians should consider PICs as a differential in cystic groin masses, even in the absence of typical risk factors.

## Introduction

Peritoneal inclusion cysts (PICs) are rare benign mesothelial proliferations, most commonly occurring in women of reproductive age with a history of prior abdominopelvic surgery or inflammation [1]. They may be unilocular or multilocular and can be attached or free within the peritoneal cavity. Although often asymptomatic, PICs can present with non-specific symptoms and are frequently diagnosed incidentally during imaging or surgery [2-4]. Their pathogenesis is thought to involve impaired peritoneal fluid reabsorption in the context of prior inflammation or adhesions [5,6].

Preoperative diagnosis can be challenging as imaging findings may overlap with other cystic pathologies. In rare cases, PICs may be found within hernia sacs, including femoral hernias, further complicating diagnosis [7]. Accurate identification is important, as femoral hernias carry a higher risk of complications and may require urgent surgical management [8].

We present a rare case of a woman in her 70s with a concurrent fatty indirect inguinal hernia and femoral hernia containing multiloculated PICs, initially mimicking a hydrocoele of the canal of Nuck on ultrasound. Only a few cases of PICs within femoral hernias have been reported, none in this age group without risk factors.

## Case presentation

A Caucasian woman in her 70s presented with a 10-month history of an asymptomatic, irreducible right groin lump measuring approximately 3-4 cm. The mass was non-tender with no overlying skin changes and demonstrated no cough impulse. She had no history of prior abdominal or pelvic surgery, endometriosis, or pelvic inflammatory disease, and reported no associated systemic or gastrointestinal symptoms.

**Figure 1:**
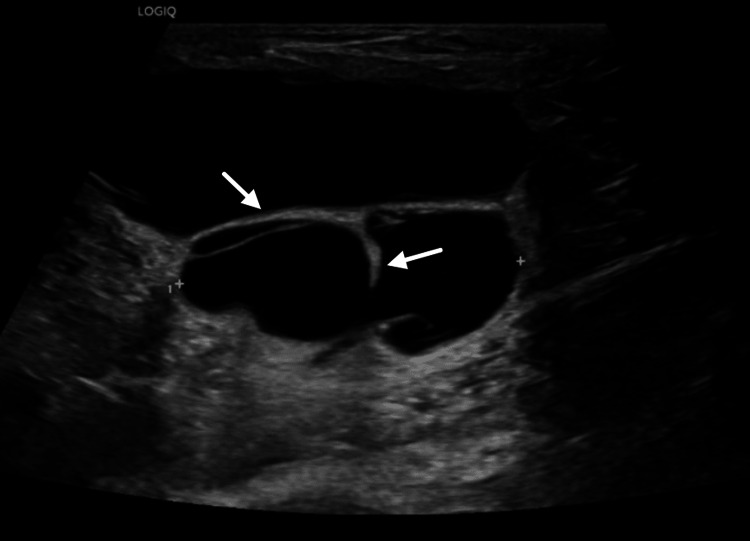
Ultrasound of the right groin demonstrating a multiloculated cystic lesion with internal septations. The appearance is consistent with a complex cystic structure and was initially interpreted as a hydrocoele of the canal of Nuck.

Ultrasound imaging demonstrated a multiloculated anechoic lesion with internal septations in the right groin (Figure 1), initially interpreted as a hydrocoele of the canal of Nuck. Given the irreducibility and diagnostic uncertainty, elective surgical exploration was undertaken.

During exploration under general anaesthesia via a Lotheissen (inguinal) approach, a small fatty indirect inguinal hernia was identified and dissected from the surrounding tissue. The defect was closed following division of the round ligament, and the deep ring was plicated.

A second cystic lesion was identified within the femoral canal. Careful dissection revealed a multiloculated cystic structure within the femoral hernia sac. On opening, straw-coloured fluid was released, with no evidence of internal communication. The lesion was completely excised near the femoral ring following confirmation, and the defect was closed with a single Prolene 1 (Ethicon, Cornelia, GA, USA) suture.

The patient made an uneventful recovery and was discharged on postoperative day one. Histopathological examination demonstrated benign multiloculated mesothelial cysts consistent with peritoneal inclusion cysts, with no evidence of malignancy. At the six-month follow-up, the patient remained well with no evidence of recurrence or postoperative complications.

## Discussion

PICs are rarely encountered within femoral hernias, with only a limited number of cases reported in the literature [9,10]. Their presence within hernia sacs represents an unusual cause of groin swelling and may contribute to diagnostic uncertainty in the evaluation of groin masses. Although atypical symptoms such as urinary disturbances have been described in cases of unusual hernia sac contents [1,11], none were observed in this patient.

In this case, the preoperative diagnosis of a hydrocoele of the canal of Nuck highlights the potential for radiological misinterpretation. Both PICs and canal of Nuck hydrocoeles can present as cystic groin masses with similar ultrasonographic features, including thin-walled, septated fluid collections [12,13]. However, these entities typically occur in different patient populations, with canal of Nuck abnormalities more commonly seen in children and younger females [14]. This discrepancy underscores the importance of correlating imaging findings with clinical context, particularly patient age and risk factors. 

The diagnostic challenge in this case was further compounded by the absence of known predisposing factors for PICs, such as prior abdominopelvic surgery, inflammation, or infection. Intraoperative findings ultimately revealed a multiloculated cystic lesion within the femoral canal, with diagnosis confirmed on histopathology. This highlights the role of surgical exploration in cases where imaging is inconclusive or misleading.

Management of PICs is typically conservative in asymptomatic cases [15]; however, the presence of an irreducible groin mass with diagnostic uncertainty justified operative intervention in this instance. The use of an inguinal approach allowed for simultaneous management of both the inguinal and femoral hernias, with an uncomplicated postoperative course.

This report has several limitations. As a single case report, the findings are inherently limited in generalisability. Additionally, no preoperative CT or MRI imaging was performed, which may have provided further characterisation of the cystic lesion prior to surgical exploration. Follow-up duration was also limited to six months, preventing assessment of long-term recurrence or postoperative outcomes.

This case emphasises the need to consider PICs as a differential diagnosis in cystic groin masses, even in older patients without typical risk factors. It also demonstrates the limitations of ultrasound imaging alone in distinguishing between rare cystic pathologies, reinforcing the role of clinical judgement and intraoperative assessment.

## Conclusions

Peritoneal inclusion cysts should be considered as a differential diagnosis in cystic groin masses, even in older patients without typical risk factors. This case highlights the potential for radiological misinterpretation, particularly when PICs mimic canal of Nuck hydrocoeles on ultrasound. Clinical correlation and intraoperative assessment remain essential for accurate diagnosis.
